# SIMBSIG: similarity search and clustering for biobank-scale data

**DOI:** 10.1093/bioinformatics/btac829

**Published:** 2022-12-23

**Authors:** Michael F Adamer, Eljas Roellin, Lucie Bourguignon, Karsten Borgwardt

**Affiliations:** Department of Biosystems Science and Engineering, ETH Zurich, 4058 Basel, Switzerland; Swiss Institute for Bioinformatics (SIB), 1015 Lausanne, Switzerland; Department of Biosystems Science and Engineering, ETH Zurich, 4058 Basel, Switzerland; Department of Health Sciences and Technology, ETH Zurich, 8008 Zurich, Switzerland; Department of Biosystems Science and Engineering, ETH Zurich, 4058 Basel, Switzerland; Swiss Institute for Bioinformatics (SIB), 1015 Lausanne, Switzerland

## Abstract

**Summary:**

In many modern bioinformatics applications, such as statistical genetics, or single-cell analysis, one frequently encounters datasets which are orders of magnitude too large for conventional in-memory analysis. To tackle this challenge, we introduce SIMBSIG (*SIM*milarity *B*atched *S*earch *I*ntegrated *G*PU), a highly scalable Python package which provides a scikit-learn-like interface for out-of-core, GPU-enabled similarity searches, principal component analysis and clustering. Due to the PyTorch backend, it is highly modular and particularly tailored to many data types with a particular focus on biobank data analysis.

**Availability and implementation:**

SIMBSIG is freely available from PyPI and its source code and documentation can be found on GitHub (https://github.com/BorgwardtLab/simbsig) under a BSD-3 license.

## 1 Introduction

With the ever-increasing amount of data produced, e.g. by genomics and next-generation sequencing, there is also demand for powerful computational tools to analyse such state-of-the-art datasets. In the UK Biobank alone, genotype data [800 000 single nucleotide polymorphisms (SNPs)] of half a million people are available (Bycroft *et al.*, 2018). This genotype dataset vastly exceeds the size of random access memory (RAM) or graphics processing units (GPU) memory of conventional computers. In general, neighbour searches in common Desktop machines can be performed in datasets up to a size of about tens of gigabytes. Therefore, algorithms and data formats, such as hdf5 files, which allow for efficient out-of-core (ooc) computations are needed to perform any analysis or data processing. In order to tackle this issue, we introduce ‘SIMBSIG = *SIM*milarity *B*atched *S*earch *I*ntegrated *G*PU’, which can efficiently perform nearest neighbour searches, principal component analysis (PCA) and K-Means clustering on central processing units (CPUs) and GPUs, both in-core and ooc. SIMBSIG bridges the gap between the functionality and ease of use of the popular scikit-learn package and the speed of current computer hardware, particularly in the ooc scenario. Comparing with the Faiss software (Johnson *et al.*, 2021), a noteworthy approach related to SIMBSIG, the lack of a scikit-learn-like interface and the relatively rigid set of distance metrics make Faiss less tailored to bioinformatics applications. Nevertheless, a future version of SIMBSIG might incorporate Faiss as an alternative backend to PyTorch.

## 2 Features

SIMBSIG is a Python package which utilizes PyTorch to ensure efficient computations and provides a high-level interface mimicking scikit-learn. At its heart, SIMBSIG comprises three modules: neighbors, clustering and decomposition. All methods accept numpy arrays or hdf5 file handles as inputs. Due to the modular structure of SIMBSIG, it will be straightforward to extend it to other data types.

We chose numpy arrays as one of our input types for the user to have a seamless transition from scikit-learn while being able to use GPU acceleration whenever needed. The hdf5 format is a hierarchical data format and provides many advantages over other tabular-data formats, which makes hdf5 file objects ideal inputs to SIMBSIG. GPU usage with SIMBSIG is optional, enabling users to apply the full flexibility offered by SIMBSIG, even without access to GPUs.

The neighbors module consists of our core routine for nearest neighbour searches in a batched, brute-force manner. This guarantees exact results with a precisely controllable memory-runtime trade-off. We implemented a batched K-Nearest Neighbour (KNN) search, where the number K of neighbours is fixed a priori, and a radius neighbour search, where all neighbours within a user-defined radius are returned. Based on this core routine, we implemented classifiers and regressors.

We use a brute-force approach only due to the infeasibility of other exact methods in this scenario while retaining most other functionality of scikit-learn such as the choice of a range of metrics including all *ℓ_p_* distances. Since, especially in genetic applications, the data are often high dimensional and subject to the curse of dimensionality, we also implemented the fractional *ℓ_p_* distance (Aggarwal *et al.*, 2001). We further allow for precomputed distance matrices or user-defined functions (‘callables’). The ‘callable’ functionality is also present in scikit-learn and provides an easy way for more sophisticated (dis-)similarity functions such as kernel functions. Kernels exist, for example, for SNP data (Wang *et al.*, 2015) and may help obtaining more accurate results.

The second core routine is batched K-Means clustering from the clustering module. The implementation is based on the fast K-Means algorithm by Sculley (2010). Similar to the NN search, we accept numpy arrays or hdf5 file handles as input, and we can cluster with respect to any *ℓ_p_* norm, the cosine distance or a ‘callable’ distance function. We additionally implemented a heuristic stopping criterion based on the maximum relative change of any cluster centre. In practice, this stopping criterion works well for the *ℓ_p_* distances; however, it may be lacking an analogue for more involved distance functions. In the current implementation, for any metrics that are not *ℓ_p_*, the relative change becomes an absolute change.

The third pillar of SIMBSIG is the decomposition module. This module provides an ooc, GPU-accelerated implementation of Halko’s approximate PCA algorithm (Halko *et al.*, 2011).

## 3 Experiments

**Fig. 1.**
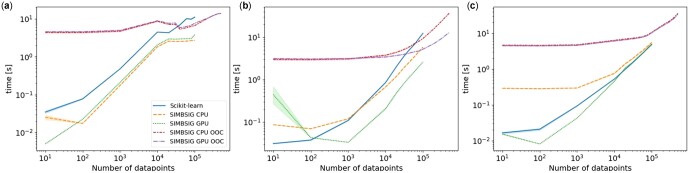
The runtime performance of SIMBSIG compared to the scikit-learn baseline averaged over 10 runs. We tested K-Means (**a**), a KNN search (**b**) and PCA (**c**). Both, in-core and ooc scenarios were computed, whereas the maximum feasible dataset size for in-core was 10^5^ datapoints. For ooc computations, there were no memory restrictions and, therefore, a wider range of dataset sizes could be explored. The batch size was 10^4^. All runs were performed on an Intel i7 10700K CPU and 32GB RAM, the GPU was an Nvidia GeForce RTX3080. Stopping criteria for K-Means were set to default values

To test accuracy, we designed an extensive set of unit tests, where we compare all possible input choices to the output of scikit-learn which we assume to be the ground truth. The speed of SIMBSIG was benchmarked on a large, artificial SNP dataset, where SNPs are encoded according to dominance assumption. We sampled ‘participants’ represented by a 10 000 dimensional vector with independent entries in {0, 1, 2}, representing 10 000 SNPs with probabilities {0.6,0.2,0.2}. In total, we sampled 500 000 such participants and stored the dataset in the hdf5 format. We tested in-memory CPU performance against scikit-learn and benchmarked ooc scenarios. A reference GPU implementation, given by cuML (Raschka *et al.*, 2020), could not use the h5py package needed to load hdf5 files. Our results are presented in Figure 1.

## 4 Results

As expected, in-core computations are faster for small datasets due to the decreased overhead of data loading compared to ooc computations. This is reflected in our results for datasets of fewer than 10^4^ points in particular. In the very small dataset regime, the neighbour searches are very efficient, therefore, any computational overhead, such as converting numpy arrays to torch tensors, or moving tensors to GPU, will result in larger runtimes. For in-core computations of larger datasets, GPU-accelerated SIMBSIG neighbour searches are almost one order of magnitude faster compared to scikit-learn. In ooc, similarity searches SIMBSIG’s GPU acceleration feature offers a significant reduction in execution time for large datasets. In the K-Means and PCA algorithms, the situation is not so clear cut. While we can still see a slight performance increase in the GPU-based PCA, the CPU counterparts seem to acquire some computational overhead from the PyTorch backend. For the PCA, execution times for all in-core methods seem to converge for larger datasets. This leads us to conclude, that SIMBSIG unfolds its full power in ooc computations and GPU-based nearest neighbour searches.

## Data Availability

All data is included in the Github repository.
